# The Effect of the Donor’s and Recipient’s Sex on Red Blood Cells Evaluated Using Transfusion Simulations

**DOI:** 10.3390/cells12111454

**Published:** 2023-05-23

**Authors:** Emmanuel Laengst, David Crettaz, Jean-Daniel Tissot, Michel Prudent

**Affiliations:** 1 Laboratoire de Recherche sur les Produits Sanguins, Transfusion Interrégionale CRS, 1066 Epalinges, Switzerland; emmanuel.laengst@itransfusion.ch (E.L.);; 2 Faculté de Biologie et de Médecine, University of Lausanne, 1011 Lausanne, Switzerland; 3 Center for Research and Innovation in Clinical Pharmaceutical Sciences, Institute of Pharmaceutical Sciences of Western Switzerland, University Hospital and University of Lausanne, 1011 Lausanne, Switzerland; 4 Institute of Pharmaceutical Sciences of Western Switzerland, University of Geneva, 1211 Geneva, Switzerland

**Keywords:** red cell concentrates, red blood cells, sex, transfusion, in vitro model, blood storage

## Abstract

The hypothesis of the potential impact of the sex of red blood cell (RBC) concentrate (RCC) donors, as well as the sex of the recipients, on the clinical outcome, is still under evaluation. Here, we have evaluated the sex impact on RBC properties using in vitro transfusion models. Using a “flask model”, RBCs from RCCs (representing the donor)—at different storage lengths—were incubated in a sex-matched and sex-mismatched manner with fresh frozen plasma pools (representing the recipient) at 37 °C, with 5% of CO_2_ up to 48 h. Standard blood parameters, hemolysis, intracellular ATP, extracellular glucose and lactate were quantified during incubation. Additionally, a “plate model”, coupling hemolysis analysis and morphological study, was carried out in similar conditions in 96-well plates. In both models, RBCs from both sexes hemolyzed significantly less in female-derived plasma. No metabolic or morphological differences were observed between sex-matched and -mismatched conditions, even though ATP was higher in female-derived RBCs during incubations. Female plasma reduced hemolysis of female- as well as male-derived RBCs, which may be related to a sex-dependent plasma composition and/or sex-related intrinsic RBC properties.

## 1. Introduction

In transplantation medicine, the sex of donors as well as recipients seems to play a role and may be a criterion of selection. Indeed, sex-dimorphisms, often related to morbidity/mortality risk of the recipient, were revealed in stem cell [1] as well as kidney [2] or heart transplantations [3,4]. In the field of blood transfusion, the female sex was considered “dangerous” because of the potential presence of anti-granulocyte or anti-HLA-antibodies in their plasma, which can initiate transfusion-related acute lung injury (TRALI) after transfusion of plasma or of platelets [5,6,7]. Nowadays, sex is not considered in the case of transfusion of red cell concentrates (RCCs). Nevertheless, some observational studies have analyzed the impact of the donor’s (as well as the recipient’s) sex on the mortality risk of the patient transfused with red blood cells (RBCs) [7]. Although in some studies, especially female-derived RBCs were stated as “risky” [8,9], others highlighted the risk being related to male products in case of sex-mismatch [10]. In contrast, others did not find an association between the donor (recipient) sex and mortality [11,12,13,14]. To shed some light on this point of contention, Zeller et al. [7] performed a meta-analysis and identified a higher mortality risk if sex-mismatched transfusions occurred. However, a potential bias of these studies is a decreased statistical strength and their differing conditions. Furthermore, many other parameters must be taken into account in such studies, notably because transfusion-related outcomes are also linked to the quality of RCCs. The latter depends on processing, storage solutions and conditions, as well as on donor characteristics [15,16].

Cold storage leads to cell lesions, including protein alterations [17,18,19,20,21], microvesiculation [22,23,24,25], morphological modifications [26,27,28] and hemolysis [15,26,29,30]. The kinetic changes in these parameters during storage vary according to blood processing [31]. Preceding this is different blood and cell properties in vivo depending on donor characteristics such as sex. Indeed, on a cellular and molecular level, several sex-specific differences have already been reported. For instance, fresh blood from men has higher levels of RBC count, hematocrit (HCT), hemoglobin (Hb) [32], and plasma antioxidant capacity [33], as well as lower osmotic fragility [33]. In contrast, women’s blood is characterized by a higher platelet count as well as lower levels of free Hb, serum iron, and cholesterol [33]. In the case of stored blood products, female-derived RCCs showed lower oxidative hemolysis and higher concentrations of ATP [34]. Regarding antioxidant defense, its mechanisms seem to differ between both sexes. On the one hand, female-derived RCCs showed higher intracellular levels of the antioxidant glutathione (GSH) [34] and a lower presence of reactive oxygen species (ROS) [33]. On the other hand, lower extracellular antioxidant power [35] and extracellular levels of the main antioxidant uric acid [35] were reported in female-derived RCCs. Another mechanism that differs between RBCs from both sexes is the production of the vasodilator nitric oxide (NO) via the estrogen receptors (ERs) [36]. Female-derived RBCs showed a higher number of potentially activated ERs α, a different phosphorylation cascade, and a resulting higher NO production compared to male-derived RBCs [36]. Not only hormonal receptors but also the impact of steroid hormones themselves on RBC properties has been studied. Female sex hormones showed positive effects on RBC membrane, their deformability, and antioxidant defense [37,38,39]. Moreover, progesterone decreased storage and osmotic hemolysis and increased ATP levels [40]. In contrast, male-derived RCCs were characterized by higher storage-linked, osmotic, and oxidative hemolyses, as well as lower post-transfusion recovery in mice [40].

Despite several studies on sex-related RBC properties, the link between the impact of the donor’s and recipient’s sex on RBC properties and their molecular impact on the recipient during transfusion is still missing. In order to gain insight into this question, the impact of the donor’s and the recipient’s sex on RBC properties was evaluated by incubating RBCs in sex-matched and sex-mismatched conditions using a previously set up transfusion model [41]. 

## 2. Materials and Methods

### 2.1. Blood Products

After donor informed consent, 14 male- and 13 female-derived RCCs in saline–adenine–glucose–mannitol (SAGM) solution (Table S1), as well as 50 male- and 50 female-derived non-filtered fresh-frozen plasma (FFP) units (Table S2), were obtained as described elsewhere [35]. Briefly, whole blood (450 ± 50 mL) was collected in 63 mL of citrate-phosphate-dextrose (CPD) anticoagulant (kit CQ32250, Fresenius Kabi, Bad Homburg, Germany). After centrifugation at 5047× *g* for 13 min at 22 °C (Roto Silenta 630 RS, Hettich, Tuttlingen, Germany), plasma and RBCs were distributed through a semi-automated pressure into sterile interconnected bags. Plasma was stored at −30 °C and RBCs in 100 mL of SAGM additive solution at 4 °C after filtration for leukoreduction. Only donors under 40 years were considered to reduce donor variability, especially related to sex hormones. Average and (median) ages, in years, of RCC/FFP donors were 29 (26)/27 (27) (“flask model”) and 28 (27)/26 (27) (“plate model”), respectively. RCCs were used for transfusion simulations after 2, 9, 29, and 42/43 days of storage.

### 2.2. In Vitro Transfusional Models

**Figure 1:**
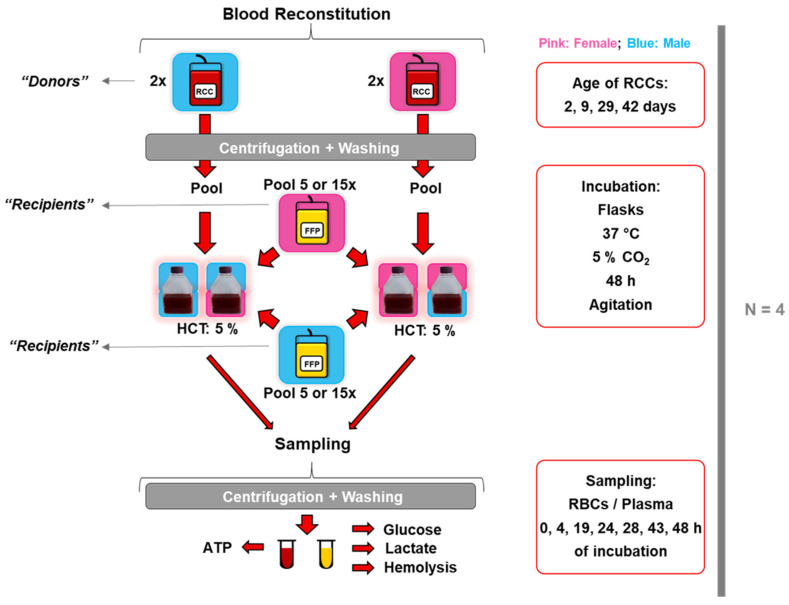
Experimental design of in vitro transfusion simulations in flasks. RBCs from male- and female-derived RCCs were extracted by centrifugation and washing steps and were then pooled (two by two). RBCs were mixed at a HCT of 5% in a sex-matched and -mismatched manner with FFP and incubated at standard conditions. At different incubations times, samples were taken and separated in RBC pellet and plasma for different analyses.

Transfusions were simulated similarly as previously described [41]. RBCs from RCCs (representing the donor) were mixed with a pool of allogeneic FFP (representing the recipient), respecting the ABO system in a sex-matched and -mismatched manner (Figure 1).

The flask model: Firstly, 16 mL from RCCs were centrifuged at 2000× *g* for 10 min at 4 °C (Rotanta 460R, Hettich, Tuttlingen, Germany), and the pellets were washed twice with NaCl 0.9% (Bichsel AG, Interlaken, Switzerland). Pellets from two RCCs were pooled and mixed with a pool of 5 or 15 FFP units at an HCT of 5% in a final volume of 140 mL in T-flasks. The “reconstituted blood” was then incubated at 37 °C (CO_2_ Water Jacketed Incubator Series II, Forma Scientific, Inc., Marietta, OH, USA) under agitation (STD 3500 Shaker, VWR, Leuven, Belgium) and 5% of CO_2_ up to 48 h. Simulations were performed with four biological replicates, each comprising two male- and two female-derived RCCs at storage days 2 to 42 (Table S1). One male- and one female-derived FFP pool were used for the first replicate, and another pair of FFP pools were used for the other three replicates (Table S2). Standard blood parameters, hemolysis, intracellular ATP, extracellular glucose, and lactate were quantified. 

The plate model: The second transfusion model was applied to increase the throughput in hemolysis analysis. RBC samples were prepared similarly, as described for the flask model, with a few variations. Without pooling, individual RBC samples were mixed with FFP pools as quadruplicates of 200 µL on 96-well plates. Two biological replicates were performed with 3 male- and 2 female-derived RCCs and 3 male- and 3 female-derived RCCs, respectively. For each replicate, a different male and female-derived FFP pool was used. Plates were incubated under the same conditions already described for the first transfusional model. Only standard blood parameters, hemolysis, and morphological changes were analyzed after incubation for 48 h.

### 2.3. Sampling

Samples of 16 mL or 5 mL (“plate model”) were taken before incubation (time point “−1”) from RCCs and from the flasks at different times of incubation. After centrifugation at 2000× *g* for 10 min at 4 °C, the supernatants were again centrifuged, whereas the RBC pellets were washed twice with NaCl 0.9%.

With respect to samples incubated in plates, plates were centrifuged at 500× *g* for 10 min in a centrifugal evaporator (GeneVac^®^ EZ-2 plus, Ipswich, UK), and the supernatants were centrifuged again for hemolysis analysis.

All samples were snap-frozen in liquid nitrogen and stored at −80 °C.

### 2.4. Standard Blood Parameters

Standard blood parameters, including RBC concentration, Hb, and HCT, were measured in RCCs, RBC pellets, and in fresh “reconstituted-blood”, samples using a blood analyzer (Sysmex KX-21N, Sysmex Digitana AG, Kobe, Japan).

### 2.5. Morphology

As described elsewhere [26], samples from the “plate model” were diluted, after 48 h incubation, in HEPA buffer without calcium and placed as quintuplicates (80,000 RBCs/well) on poly-L-ornithine-coated 96-well plates. After acquisition with a digital holographic microscope (DHM) (DHM^®^ T1000, Lyncee Tec SA, Lausanne, Switzerland), the DHM images were treated with the software CellProfiler Analyst (2.0 r11710, Broad Institute, Main St., Cambridge, MA, USA), allowing the quantification of different RBC shapes as previously described [26,41].

### 2.6. Intracellular ATP Quantification 

Washed RBC pellets were thawed and deproteinized with the Deproteinizing-Sample-Preparation Kit (BioVision, Milpitas, CA, USA) and treated with the ATPlite-Luminescence-ATP-Detection-Assay-System kit (PerkinElmer, Groningen, The Netherlands). Luminescence was measured on duplicates with a microplate reader (Spectramax M3, Molecular Devices, Silicon Valley, CA, USA) at a 1000 ms integration time.

### 2.7. Extracellular Glucose and Lactate Quantification

Extracellular glucose and lactate were quantified via colorimetry using a Glucose Assay Kit (BioChain^®^, Newark, CA, USA) at 620 nm and the Lactate Colorimetric Assay Kit II (BioVision, Milpitas, CA, USA) at 450 nm.

### 2.8. Hemolysis Rate

The determination of the hemolysis rate was based on Harboe’s method, as previously described [26,42]. Briefly, free Hb was quantified in supernatants at 415 nm, including corrections at 380 nm and 450 nm with a NanoDrop 2000c (Thermo Scientifc, Wilmington, DE, USA). Hemolysis rate was calculated as follows.

$$Free\;Hb\;\left(g/L\right)=\left(167.2\times A_{415}-83.6\times A_{380}-83.6\times A_{450}\right)\times 0.01\times dilution\;factor$$

$$Hemolysis\;rate\;\left(\%\right)=\frac{free\;Hb\;(g/L)\times \left(100-HCT\;\left(\%\right)\right)}{total\;Hb\;(g/L)}$$

For the “plate model”, a microplate reader (Spectramax M3, Molecular Devices, Silicon Valley, CA, USA) was used.

### 2.9. Data Treatment and Statistics

Graphs and statistics were carried out with the software GraphPad Prism 6 (GraphPad Software Inc., San Diego, CA, USA). Statistical analyses were based on multiple comparisons with Two-Way ANOVA using a significance threshold of *p* ≤ 0.05. 

## 3. Results

### 3.1. Sex Did Not Influence Hemolysis during Storage

**Figure 2:**
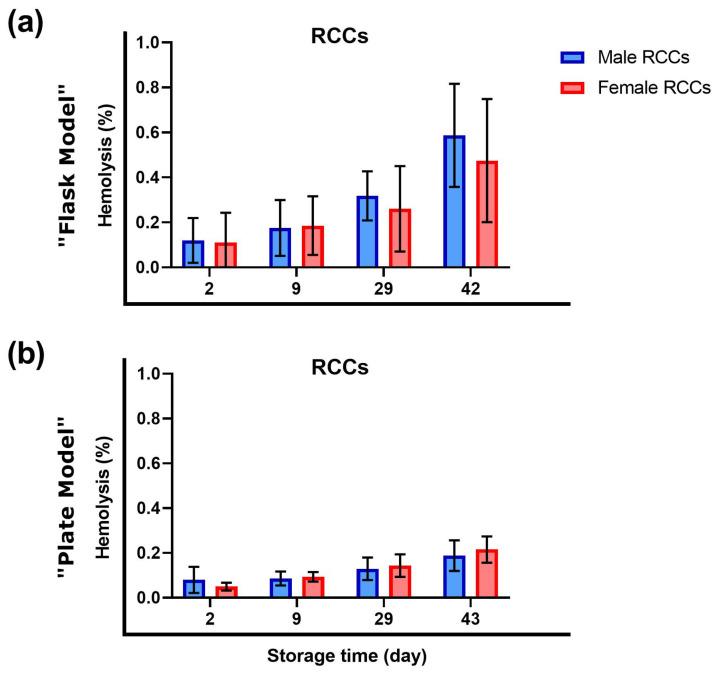
Hemolysis of RBCs in RCCs before transfusion simulations. RCCs either designated for (**a**) the “flask model” assay or for (**b**) the “plate model” experiment were stored at 4 °C, up to 43 days. Hemolysis rates were quantified, according to Harboe’s method [26,42], at different storage days. (**a**) N = 6; (**b**) N = 6 (male) or 5 (female). Values are presented ± standard deviation.

The evolution of hemolysis was monitored in RCCs stored at standard conditions that were designated for transfusion simulation experiments (Figure 2). Hemolysis increased during storage time as expected [26,41,43]. A different hemolytic profile between male- and female-derived RCCs could not be detected.

### 3.2. The Sex Influences Hemolysis of Red Blood Cells during Transfusion Simulations 

**Figure 3:**
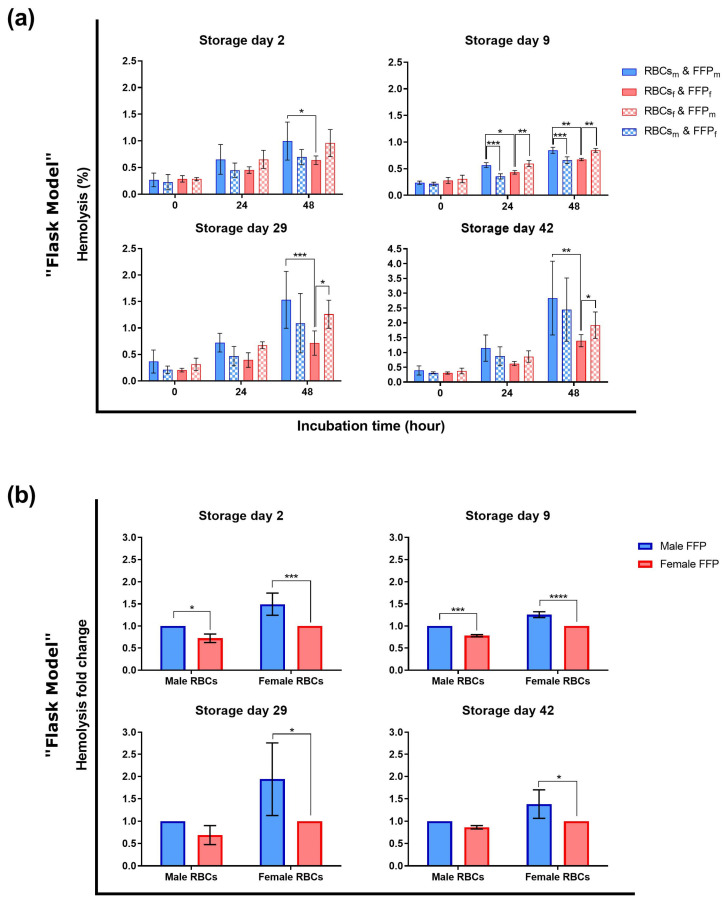
Hemolysis of RBCs during transfusion simulations in vitro (“flask model”). A pool of 2 RBC pellets from male (m) or female-derived (f) RCCs (stored at 4 °C, up to 42 days) were incubated in a sex-(mis)matched manner with male- (m) and female-derived (f) FFP pools up to 48 h (37 °C, 5% CO_2_ under agitation). (**a**) hemolysis rates and (**b**) hemolysis fold changes were quantified, according to Harboe’s method [26,42]. N = 4 (at day 9, N = 3). *p*-values: * < 0.05, ** < 0.01, *** < 0.001, **** < 0.0001. Values are presented ± standard deviation.

In order to analyze the impact of the sex in a transfusion context, RBCs were incubated with plasma in a sex-matched and -mismatched manner and hemolysis was quantified (Figure 3a). As expected [26,41,43], hemolysis increased with storage and incubation time. Incubated male-derived RBCs showed higher hemolysis than female-derived ones considering sex-matched, especially at the end of incubation at all storage days (day 29 *p* < 0.001). While female-derived RBCs hemolyzed significantly less when incubated in female-derived plasma at storage days 9, 29, and 42, male-derived RBCs in female-derived plasma significantly showed this characteristic only at day 9. 

In order to counteract individual hemolysis variations, hemolysis fold change was calculated between sex-matched and -mismatched conditions (Figure 3b). During the whole storage time, female-derived RBCs incubated in women’s plasma hemolyzed significantly less than those incubated in plasma from male donors. This difference between sex-matched and -mismatched conditions was particularly pronounced at storage day nine (*p* < 0.0001). Regarding RBCs from male donors, female-derived plasma significantly reduced hemolysis compared to male-derived plasma, but only at storage days two (*p* < 0.05) and nine (*p* < 0.001).

**Figure 4:**
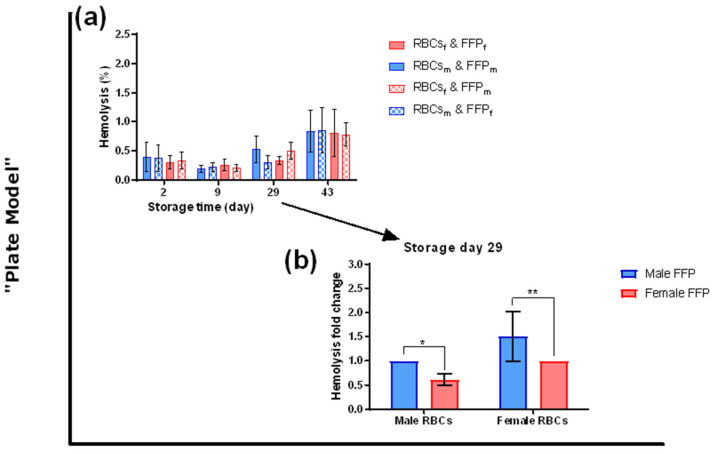
Hemolysis of RBCs during transfusion simulations in vitro (“plate model”). RBC pellets from male- (m) or female-derived (f) RCCs (stored at 4 °C, up to 43 days) were incubated in a sex-(mis)matched manner with male- (m) and female-derived (f) FFP pools up to 48 h (37 °C, 5% CO_2_ under agitation). (**a**) hemolysis rates and (**b**) hemolysis fold changes were quantified, according to Harboe’s method [26,42]. N = 6 (male) and 5 (female). *p*-values: * < 0.05, ** < 0.01. Values are presented ± standard deviation.

The effect of plasma donor’s sex on RBCs’ hemolytic properties was further evaluated with additional RBC and plasma mixes in 96-well plates after 48 h incubation (“plate model”) (Figure 4a). Hemolysis levels of incubated RBCs increased with storage time and showed high individual variations. No differences were observed between male- and female-derived RBCs or between sex-matched and -mismatched conditions. However, hemolysis fold change analysis (Figure 4b) revealed, for both male- and female-derived RBCs, a hemolysis reduction when incubated in women’s plasma (male *p* < 0.05; female *p* < 0.01). 

### 3.3. Similar Metabolic Activity during Transfusion Simulations 

**Figure 5:**
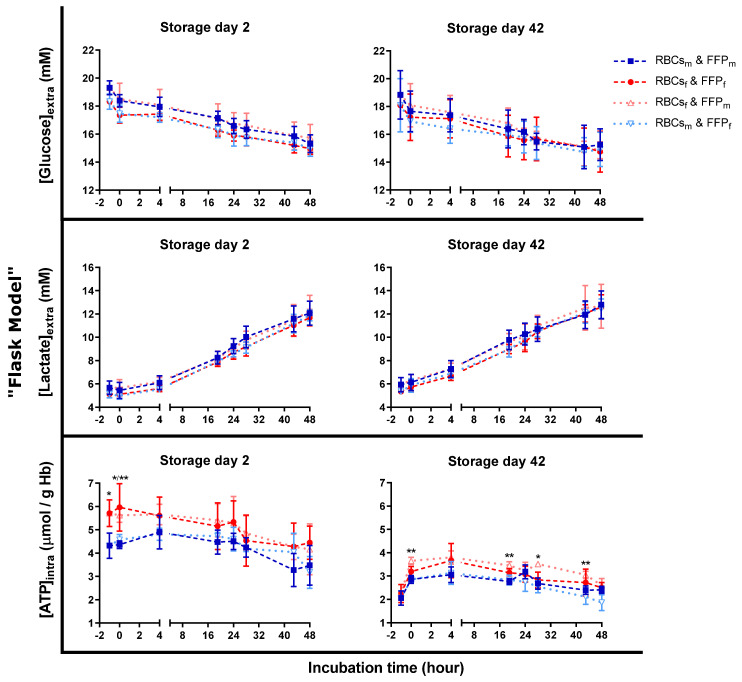
Quantification of RBC metabolites during transfusion simulations in vitro (“flask model”). RBCs from male- (m) and female-derived (f) RCCs (stored at 4 °C, up to 42 days) were incubated in a sex-(mis)matched manner with male- (m) or female-derived (f) FFP pools up to 48 h (37 °C, 5% CO_2_ under agitation). Metabolites were quantified before (“−1”) incubation and at different incubation times in the supernatants (extracellular) or in the RBC pellets (intracellular) after washing with 0.9% NaCl. N = 4. *p*-values: * < 0.05, ** < 0.01. Values are presented ± standard deviation.

To evaluate the impact of sex on RBC metabolism, extracellular glucose and lactate levels, as well as intracellular ATP concentrations, were quantified (Figure 5). No significant differences were reported. Nevertheless, glucose levels were slightly higher in FFP from male donors. However, glucose decrease was similar in all conditions on both storage days. Lactate concentration increase correlated with glucose levels and was similar in all conditions.

Intracellular ATP levels were higher in female-derived RCCs than in RCCs from men at storage day two. This difference increased at the beginning of incubation. Afterward, ATP levels of male-derived RBCs continued to increase up to 4 h of incubation while those of female-derived RBCs started to decrease. At 4 h, the ATP difference was lost between both sexes. At storage day 42, the ATP sex difference between RCCs was offset (data at time “−1”). On this day, ATP levels were generally lower in all incubation conditions compared to day two, despite a clear rejuvenation capacity [41]. Differences between male- and female-derived RBCs were induced during incubation and maintained. The highest ATP levels were observed in female-derived RBCs incubated in male-derived plasma, and the lowest was detected in male-derived RBCs and plasma. However, no differences were observed between sex-matched and sex-mismatched conditions during both storage days. The ATP differences were intrinsic to the RBCs.

### 3.4. Sex Did Not Affect Red Blood Cell Morphology during Transfusion Simulations

**Figure 6:**
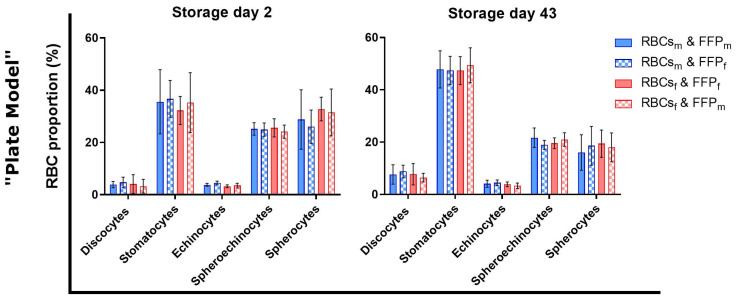
Morphology analyses of RBCs during transfusion simulations in vitro (“plate model”). Samples from RBCs incubated for 48 h in FFP at 37 °C were diluted in HEPA buffer and analyzed with a DHM. Proportions of the RBC shapes were estimated with CellProfiler^®^. N = 6 (male) or 5 (female). Values are presented ± standard deviation.

As seen in Figure 3 and Figure 4, hemolysis rates were influenced by sex. Because morphologic changes of RBCs precede hemolysis, we analyzed if sex-dependent morphology alterations appeared. Thus, the morphology of RBCs was evaluated with a DHM after 48 h of incubation in 96-well plates (“plate model”) (Figure 6). At storage day two, the proportions of discocytes were already low, and those of spherocytes and spheroechinocytes were more than 50%. Incubated long-stored RBCs showed a similar profile. Interestingly more stomatocytes but fewer spherocytes appeared on day 43 compared to day 2. Contradictory to hemolytic parameters, for both days, no sex differences were observed, related neither to intrinsic RBC properties nor to incubation conditions.

## 4. Discussion

### 4.1. Hemolysis in Red Cell Concentrates 

Hemolysis occurs when the RBC membrane is weakened and disrupted [25,44]. Causes of weakening can be the reduced activity of energy and antioxidant metabolism increasing oxidative stress [25]. Oxidative reactions cause inter alia weakening of the cytoskeleton-membrane link by alteration of lipids and proteins that are evacuated by microvesiculation [18,25]. An excess of microvesiculation additionally favors hemolysis [25].

In contrast to other publications [29,40,45], we did not detect lower hemolysis levels in stored female-derived RCCs compared to their male counterpart (Figure 2). First, the number of donors could be too small since this difference was reported in important cohorts [29]. Second, our donor cohort could have been “too young” (mostly < 35 years). Indeed, Kanias et al. [29] observed a maximal sex-dependent storage hemolysis difference at the age of around 40 years because of male-derived RBCs.

Other studies concluded that sex does not influence storage hemolysis [33]. In our case, hemolysis in RCCs showed increased individual variations independent of the donor’s sex. These hemolytic variations also depend on other factors, such as ethnicity [34]. Moreover, in the present study, the menstrual cycle or the intake of contraceptives was not considered. However, hormone variations can influence RBC deformability and membrane fluidity [32,37]. At this stage, it is difficult to determine the reasons for similar hemolysis in this case, as it is a multiplex parameter depending on genetic and metabolic factors.

### 4.2. Hemolysis during Transfusion Simulations

Despite the absence of sex-related differences in the RCCs, hemolysis of RBCs incubated in plasma at 37 °C differed between both sexes (Figure 3 and Figure 4). Lower hemolysis during cold storage, mainly due to temperature, could have covered sex differences in contrast to more pronounced hemolysis at 37 °C, as expected [41].

#### 4.2.1. Metabolic and Morphological Data Cannot Explain the Hemolytic Differences

The higher initial glucose levels in male-derived FFP were due to processing (Figure 5). Since female donors have a lower HCT than men, a higher plasma content is mixed with the glucose-containing anticoagulant CPD solution. Otherwise, glucose consumption and lactate production were similar for both sexes, as reported elsewhere [46].

ATP differences were present but seemed to be intrinsic to male- and female-derived RBCs with only a small plasma-type effect (Figure 5). ATP levels were mainly the highest in female-derived RBCs incubated in male-derived plasma and the lowest in male-derived RBCs in male plasma. At the same time, these conditions showed a similar hemolysis rate (Figure 3a). Hence, the observed ATP differences do not explain the hemolytic variations. The initial ATP difference before and at the beginning of incubation on day two may just be a relic of physiological blood nature, independent of the incubation conditions (Figure 5). Women’s blood products are characterized by a lower oxygen saturation [47]. It may favor the liberation of the glycolytic enzymes from band 3 and hence, boost glycolysis and ATP production compared to male-derived RBCs [48,49]. The ATP difference during storage was probably offset by oxygen saturation increase [50] and equilibration between both sexes via the semi-permeable RCC bags. Nevertheless, at storage day 42, ATP differences appeared for male- and female-derived RBCs in male plasma during incubations. Consequently, this observation is independent of oxygenation status, particularly as flasks were incubated in the same oxygenated environment with a regulated CO_2_ concentration. It means that the main contributor of ATP difference must be intrinsic to the RBCs with a slight contribution of the plasma type. The ATP difference may depend on how male- and female-derived RBCs manage the exposure to oxidative stress. Already in RCCs, before incubation, male-derived RBCs lose higher amounts of the main antioxidant urate than female ones [35]. This leakage may be due to a re-equilibration of intracellular urate concentrations with lower extracellular concentrations caused by the dilution of residual plasma urate with the additive solution [43]. In order to compensate for this loss, glycolysis may be strongly rerouted to the pentose phosphate pathway (PPP) at the expense of ATP production. Additionally, ATP may be consumed to support the recycling of another antioxidant, the GSH. Nevertheless, as already mentioned, differing ATP levels cannot explain the sex-dependent hemolytic profiles.

The similitude of the morphological profiles may be due to the deselection of apoptotic RBCs that may have already hemolyzed during washing steps before incubation. Moreover, since a huge part of RBCs already had a spheroid shape after 48 h of incubation, subtle effects of the sex could not have been detected anymore. In order to investigate further the impact, cell morphology could be analyzed earlier, before these altered conditions. The low discocyte quantity and the increased stomatocyte population, also observed previously [41], may be due to bioactive lipids. Indeed, these molecules can accumulate in warm-incubated plasma and integrate into the RBC membrane [51,52]. Additionally, the ionic composition of the HEPA buffer used for RBC dilution for DHM analyses may have affected the RBCs despite recommendations for in vitro analyses [53]. Since RBCs were already weakened by 48 h incubation at 37 °C, they may not have been able to adapt to these new conditions.

#### 4.2.2. Plasma Composition Could Explain the Hemolytic Profile

Sex differences may not only be intrinsic to RBCs but also depend on the plasma type. Plasma is diluted in RCCs, which dilutes plasma molecules and their potential impact on RBCs; incubated RBCs were fully exposed to plasma.

The obvious plasma differences between men and women, the sex hormones, could influence RBC properties. Indeed, estrogen and progesterone are supposed to favor deformability [38,39] and reduce ROS formation by increasing the activation of the CuZn superoxide dismutase [54,55], which could delay hemolysis [15]. Furthermore, female-derived RBCs seem to have more activated estrogen receptors (α-ERs), resulting in higher NO levels that may also improve deformability [32,36]. 

The different hemolytic profiles could also originate from other factors present in the plasma, such as proteins [56] and other lipids [57,58]. For instance, sex-dependent levels of eicosanoid derivatives, sphingomyelin (SM), and phosphatidylcholines (PCs) were detected in plasma [57,58]. These molecules can be implicated in the membrane structure but also in signal transduction and protein recruitment to the membrane [59,60,61,62]. SM molecules were elevated in female-derived plasma. The occurrence of SMs and other lipids seems to be related to female sex hormones [63,64,65]. An increased SM level in plasma could improve the stability of the RBC membrane or modify its composition. This hypothesis could explain a higher resistance of RBCs in female-derived plasma against hemolysis. 

In summary, different protein and lipid profiles of male- and female-derived plasma may have differently affected the stability, deformability as well as hemolytic tendencies of RBCs. These observations provide a further source of investigation.

### 4.3. Limits of the Study

The FFP used for our study contained approximately four times higher glucose levels than physiologic plasma, which may have repercussions on protein glycation [66]. The donor age was limited to 40 years. We intended to reduce donor variability related to age because hemolysis differences, amongst other differing parameters, were observed regarding RBCs from pre- and postmenopausal women [33] as well as regarding the age of men [29]. However, we did not consider the hormonal status of the donors (menstrual cycle or contraceptives), which may have influenced RBC properties [32,37]. In addition, measured metabolites give an idea of RBC metabolism but are not sufficient to understand the impact of sex on metabolism.

## 5. Conclusions

Sex-dependent hemolytic differences were observed during in vitro transfusion simulations with lower hemolysis in the presence of female-derived plasma. Factors leading to increased hemolysis could not be clearly determined: glucose intake and lactate production were similar between both sexes. Nevertheless, intracellular ATP levels differed between both males and females independently of sex-(mis)matching. On a cellular level, a considerable part of RBCs progressed to irreversible shapes. Sex differences were not detected: they should have been evaluated at an earlier stage of incubation. The investigation of the pathways of other metabolites would be necessary to understand the conduction of metabolic fluxes as well as their impact on a molecular and cellular level. Additionally, to discriminate better if observations originate from intrinsic properties of RBCs or from sex-dependent factors in the plasma, incubations with a “sex-neutral” medium could be tested, too.

In this study, sex differences were observed with “young”- and “middle-aged” RCCs. This is of interest because most RCCs are transfused around 20 days of storage [67,68].

## Data Availability

Data supporting reported results or other information will be available on demand.

## Supplementary Materials

The following supporting information can be downloaded at: https://www.mdpi.com/article/10.3390/cells12111454/s1, Table S1: Standard blood parameters of individual RCCs and donor information; Table S2: Standard blood parameters of individual FFP units and donor information.
