# Supplementary material for: The Effect of the Donor’s and Recipient’s Sex on Red Blood Cells Evaluated Using Transfusion Simulations
Source: Cells. 2023 May 23;12(11):1454. doi: 10.3390/cells12111454 (PMC10252512; doi:10.3390/cells12111454)
Supplement: Supplementary file 1 [file cells-12-01454-s001.zip › cells-2299008-supplementary.pdf]

Supplementary Data

Table S1. Standard blood parameters of individual RCCs and donor information

"Flask Model" (4 replicates)

Storage Day	2	42	2	42	2	42	2	42	2	42	2	42	2	42	2	42
RCC	***1 (♂)***		***2 (♂)***		***3 (♀)***		***4 (♀)***		***5 (♂)***		***6 (♂)***		***7 (♀)***		***8 (♀)***	
**Hb (g/L)**	/	/	/	/	/	/	/	/	208	208	208	204	176	186	186	190
**HCT (%)**	/	/	/	/	/	/	/	/	60	63	61	62	55	57	56	57
**Hemolysis (%)**	/	/	/	/	/	/	/	/	0.06	0.26	0.08	0.48	0.05	0.18	0.05	0.28
**Donor age (years)**	27		20		24		24		39		25		32		34	
**ABO RhD**	O+		O+		O+		O+		A+		A+		A+		A+	

Storage Day	2	42	2	42	2	42	2	42	2	42	2	42	2	42	2	42
RCC	***9 (♂)***		***10 (♂)***		***11 (♀)***		***12 (♀)***		***13 (♂)***		***14 (♂)***		***15 (♀)***		***16 (♀)***	
**Hb (g/L)**	198	194	206	210	180	182	192	200	206	204	212	210	198	198	182	182
**HCT (%)**	61	62	63	66	53	56	56	60	59	62	60	64	58	59	58	60
**Hemolysis (%)**	0.05	0.71	0.05	0.21	0.05	0.25	0.07	0.62	0.29	0.65	0.20	0.59	0.38	0.95	0.02	0.26
**Donor age (years)**	38		35		21		35		22		25		34		26	
**ABO RhD**	A+		A+		A+		A+		A+		A+		A+		A+	

"Plate Model" (2 replicates)

Storage Day	2	43	2	43	2	43	2	43	2	43	2	43	2	43	2	43	2	43	2	43	2	43
RCC	***1 (♂)***		***2 (♂)***		***3 (♂)***		***4 (♀)***		***5 (♀)***		***6 (♂)***		***7 (♂)***		***8 (♂)***		***9 (♀)***		***10 (♀)***		***11 (♀)***	
**Hb (g/L)**	230	230	216	210	212	214	178	182	190	190	188	210	188	198	194	202	180	184	204	184	184	192
**HCT (%)**	61	68	62	67	61	72	53	57	57	58	58	67	59	61	60	67	56	59	61	60	58	62
**Hemolysis (%)**	0.19	0.24	0.08	0.19	0.09	0.29	0.08	0.26	0.05	0.29	0.04	0.11	0.04	0.18	0.04	0.12	0.05	0.16	0.04	0.22	0.03	0.15
**Donor age (years)**	27		33		27		23		35		38		19		34		20		26		31	
**ABO RhD**	O+		A+		A+		O-		A+		A+		O+		A+		A+		O+		A+	

Hemoglobin (Hb), the hematocrit (HCT) and hemolysis were measured in the RCCs that were stored up to 43 days at 4 °C under blood-bank conditions. Furthermore, the donor age and ABO RhD are indicated.

Table S2. Standard blood parameters of individual FFP units and donor information

"Flask Model" (4 replicates)

Replicate 1

FFP	Blood group	Donor age (year)	Storage (day)
1 *(♂)*	AB+	25	90
2 *(♂)*		25	64
3 *(♂)*		25	62
4 *(♂)*		33	44
5 *(♂)*		27	28
1 *(♀)*		28	63
2 *(♀)*		27	64
3 *(♀)*		22	64
4 *(♀)*		27	65
5 *(♀)*		27	64

Replicates 2-4

FFP	Blood group	Donor age (year)	Storage (day)
1 *(♂)*	AB+	24	127
2 *(♂)*		30	133
3 *(♂)*		26	127
4 *(♂)*		22	135
5 *(♂)*		24	128
6 *(♂)*		28	44
7 *(♂)*		18	37
8 *(♂)*		24	37
9 *(♂)*		20	37
10 *(♂)*		27	132
11 *(♂)*		38	44
12 *(♂)*		28	37
13 *(♂)*		25	37
14 *(♂)*		34	33
15 *(♂)*		37	44
1 *(♀)*		31	161
2 *(♀)*		34	135
3 *(♀)*		23	135
4 *(♀)*		37	127
5 *(♀)*		27	134
6 *(♀)*		32	135
7 *(♀)*		26	44
8 *(♀)*		18	42
9 *(♀)*		21	41
10 *(♀)*		37	33
11 *(♀)*		27	41
12 *(♀)*		25	37
13 *(♀)*		19	35
14 *(♀)*		31	44
15 *(♀)*		27	41

"Plate Model" (2 replicates)

Replicate 1				***Replicate 2***			
**FFP**	**ABO RhD**	**Donor age (year)**	**Storage (day)**	**FFP**	**Blood group**	**Donor age (year)**	**Storage (day)**
1 (♂)	AB+	31	495	***1 (♂)***	AB+	36	49
2 (♂)		25	495	***2 (♂)***		28	86
3 (♂)		22	467	***3 (♂)***		22	86
4 (♂)		21	467	***4 (♂)***		27	53
5 (♂)		25	467	***5 (♂)***		24	86
6 (♂)		28	467	***6 (♂)***		24	64
7 (♂)		25	467	***7 (♂)***		19	91
8 (♂)		33	443	***8 (♂)***		36	126
9 (♂)		27	443	***9 (♂)***		20	48
10 (♂)		24	408	***10 (♂)***		28	86
11 (♂)		30	408	***11 (♂)***		30	64
12 (♂)		24	408	***12 (♂)***		31	59
13 (♂)		24	328	***13 (♂)***		34	64
14 (♂)		20	328	***14 (♂)***		30	48
15 (♂)		38	328	***15 (♂)***		33	51
1 (♀)		27	495	***1 (♀)***		27	49
2 (♀)		19	495	***2 (♀)***		30	64
3 (♀)		36	495	***3 (♀)***		25	57
4 (♀)		18	495	***4 (♀)***		18	63
5 (♀)		21	495	***5 (♀)***		35	91
6 (♀)		28	467	***6 (♀)***		28	56
7 (♀)		27	467	***7 (♀)***		34	63
8 (♀)		22	467	***8 (♀)***		19	61
9 (♀)		27	467	***9 (♀)***		26	58
10 (♀)		23	408	***10 (♀)***		31	43
11 (♀)		18	328	***11 (♀)***		22	63
12 (♀)		21	328	***12 (♀)***		32	51
13 (♀)		27	328	***13 (♀)***		22	48
14 (♀)		19	328	***14 (♀)***		18	86
15 (♀)		31	328	***15 (♀)***		22	50

Donor sex, blood group and storage time (at -30 °C) of used FFP units are indicated.
